# Depression, anxiety, stress and ways to overcome them among healthcare workers during the first wave of the COVID-19 pandemic in Russia

**DOI:** 10.1192/j.eurpsy.2026.11014

**Published:** 2026-07-31

**Authors:** V. I. Rozhdestvenskiy, V. V. Titova, I. A. Gorkovaya, D. O. Ivanov, Y. S. Aleksandrovich

**Affiliations:** Department of Psychosomatics and Psychotherapy, Saint Petersburg State Pediatric Medical University, Saint Petersburg, Russian Federation

## Abstract

**Introduction:**

During the first wave of the COVID-19 pandemic in Russia, healthcare workers (doctors, nurses, etc.), according to numerous scientific studies, experienced depression, anxiety, and stress, which were manifestations of adjustment disorder. To cope with these symptoms, healthcare workers resorted to various coping strategies, which had varying degrees of effectiveness in the context of the pandemic and in some cases were even completely ineffective.

**Objectives:**

The study aimed to identify the relationships between affective disorders (depression, anxiety), stress, and coping strategies among Russian medical professionals during the first wave of the COVID-19 pandemic.

**Methods:**

Data were collected between May and July 2020 using a Google Form developed by the authors. The study sample consisted of 45 healthcare professionals residing in Russia. Coping strategies were assessed with the COPE-60 inventory, while levels of depression, anxiety, and stress were measured using the DASS-21 scale. Both instruments were adapted for use in the Russian context.

**Results:**

We found that depression has positive correlations with the coping strategies ‘behavioural avoidance’ (r_s_ = 0.532, p < 0.01), “restraint” (r_s_ = 0.466, p < 0.01), ‘use of “sedatives”’ (r_s_ = 0.370, p < 0.01) and ‘acceptance’ (r_s_ = 0.311, p < 0.05); anxiety is positively correlated with ‘denial’ (r_s_ = 0.409, p < 0.01), ‘turning to religion’ (r_s_ = 0.404, p < 0.01), ‘behavioural avoidance of the problem’ (r_s_ = 0.365, p < 0.05) and ‘use of “sedatives”’ (r_s_ = 0.309, p < 0.05); stress has positive correlations with ‘turning to religion’ (r_s_ = 0.331, p < 0.05), ‘behavioural avoidance of the problem’ (r_s_ = 0.454, p < 0.01), “restraint” (r_s_ = 0.380, p < 0.01) and ‘use of emotional social support’ (r_s_ = 0.505, p < 0.01).

**Conclusions:**

During the first wave of the COVID-19 pandemic in Russia, all coping strategies used by healthcare workers were ineffective in combating depression, anxiety and stress, and even exacerbated them. The most ‘dangerous’ coping strategy was ‘behavioural avoidance of the problem.’ This suggests that acknowledging their helplessness in the pandemic situation was the most painful experience for medical workers, causing strong negative emotions. It is characteristic that seeking emotional support from others tends to intensify stress among healthcare professionals. It is essential to develop new coping strategies that can effectively support healthcare professionals in managing psychologically challenging situations during epidemics.

**Disclosure of Interest:**

None Declared

